# MultiFixRadSoft: A Comprehensive Tool for Primary Relative Radiometric Scale Realization in Radiation Thermometry

**DOI:** 10.3390/s26082489

**Published:** 2026-04-17

**Authors:** Mehtap Ertürk, Mevlüt Karabulut, Ömer Faruk Kadı, Can Gözönünde, Patrik Broberg, Åge Andreas Falnes Olsen, Humbet Nasibli

**Affiliations:** 1 National Metrology Institute, The Scientific and Technological Research Council of Türkiye (TÜBİTAK-UME, Ulusal Metroloji Enstitüsü), Gebze 41470, Türkiye; omerfaruk.kadi@tubitak.gov.tr (Ö.F.K.); can.gozonunde@tubitak.gov.tr (C.G.); 2 Department of Physics, Gebze Technical University, Gebze 41400, Türkiye; mevlutk@gtu.edu.tr; 3 RISE—Research Institutes of Sweden, 504 62 Borås, Sweden; patrik.broberg@ri.se; 4 JV—Justervesenet, 2007 Kjeller, Norway; aao@justervesenet.no

**Keywords:** radiation thermometry, relative primary radiometry, uncertainty, *MeP*-K, eutectic fixed-points, Sakuma–Hattori equation, HTFPs

## Abstract

This paper presents a practical implementation of relative primary radiation thermometry (RPRT) together with MultiFixRadSoft, an open-source software package developed in accordance with the Mise-en-Pratique for the kelvin (*MeP*-K) for realization of the thermodynamic temperature scale and uncertainty evaluation under the new definition of the kelvin. The software enables realization of temperature scales using ITS-90 metal fixed points as well as metal–carbon and metal–carbide–carbon eutectic high-temperature fixed points (HTFPs) for both radiation thermometers and radiometers. It incorporates automated routines for melting plateau analysis, including determination of the point of inflection, liquidus point, and melting range, together with correction modules for size-of-source effect, detector nonlinearity, emissivity, and temperature drop. Validation is demonstrated through experimental realization using six fixed points (Cu, Fe–C, Co–C, Pd–C, Ru–C, and WC–C) and a linear radiation thermometer. The software also supports ITS-90 extrapolation procedures and flexible calibration schemes (*n* = 1 to *n* ≥ 3), with automated Sakuma–Hattori fitting and full uncertainty propagation compliant with *MeP*-K requirements. The results show excellent agreement with manual analyses and published data, confirming the correctness of the implemented algorithms. By integrating data processing, scale realization, and uncertainty analysis within a unified and transparent framework, MultiFixRadSoft provides a robust and accessible tool for traceable radiometric thermometry, supporting emerging NMIs and industrial laboratories while promoting the wider adoption of primary thermodynamic temperature realization methods.

## 1. Introduction

Temperature sensing in the high-temperature regime (above 1200 K) is predominantly performed using non-contact thermometric techniques, although contact-based methods remain applicable in certain specialized cases. Within this domain radiation thermometry offers the lowest measurement uncertainty, combined with robustness, rapid response and remote detection. Therefore, in the temperature scales defined by the International Committee for Weights and Measures (CIPM)—most notably the latest one, the International Temperature Scale of 1990 (ITS-90) [1]—SI-traceability (International System of Units) of temperature measurements in this range is established through radiation thermometry methods.

With the recent redefinition of the SI units [2] in terms of fundamental physical constants, the unit of temperature—the kelvin (symbol: K)—has been redefined on the basis of the Boltzmann constant [3,4]. This shift marks a departure from the former reliance on the triple point of water as the defining reference. Instead, the kelvin was established through a fixed numerical value of the Boltzmann constant [5]. Radiation thermometry is essentially governed by the Planck law relating spectral radiance density from a blackbody to its thermodynamic temperature, as given by:

(1) $$L_{\lambda }\left(T\right)=\frac{2hc^{2}}{\lambda^{5}}\frac{1}{\exp\left(\frac{hc}{\lambda k_{B}T}\right)-1}$$

where $L_{\lambda }\left(T\right)$—spectral radiance at wavelength λ and absolute temperature T, h—Planck’s constant, c—speed of light in vacuum, λ—wavelength, $k_{B}$—Boltzmann constant. By precisely measuring the radiance value $L_{\lambda }\left(T\right)$, the corresponding thermodynamic temperature T can be extracted according to Equation (1), which only depends on other SI base units (mass via h, and time/frequency via c and λ) and hence represents a primary measurement of the kelvin.

In this context, the *Mise-en-Pratique* for the kelvin (*MeP*-K) has been updated with recommended routes to implement traceable temperature measurements, which go beyond the previous scale definition [6,7,8]. Within the aforementioned temperature range, it describes several methods for determining thermodynamic temperature [9,10]. These methods include absolute and Relative Primary Radiometric Thermometry (RPRT), as well as the approximation of thermodynamic temperature by $T_{90}$ [11,12,13].

An absolute measurement must be performed using a radiometer observing an isothermal cavity with a known emissivity over a precisely determined solid angle, within a narrow spectral band for which the absolute spectral responsivity of the instrument is accurately characterized. Currently, absolute primary radiometric measurements can be realized using four distinct optical configurations; namely, the power, irradiance, hybrid, and radiance methods, as described by Anhalt and Graham [14]. These techniques are accepted as the primary level routines and were improved and implemented within several EU-funded research projects, including InK, InK 2, and Real-K [15,16,17].

The *MeP*-K also enables relative primary radiometry as a complementary means of realizing the radiation temperature scale based on thermodynamic temperature in the high-temperature range [8,9,12]. The purpose is to simplify measurements by replacing explicit photometric traceability with thermometric traceability. This approach relies on a set of reference fixed points, whose reference thermodynamic temperatures and associated uncertainties have been assigned by means of absolute radiometry [18,19]. Depending on the number and selection of these reference fixed points, appropriate interpolation or extrapolation procedures are applied to construct the temperature scale, as discussed in Section 2.5.

In recent years—through the aforementioned EU projects and in collaboration with the Consultative Committee for Thermometry Working Group on Non-Contact Thermometry (CCT-WG-NcTh) and the broader radiation thermometry community—a comprehensive set of reference fixed points with internationally accepted thermodynamic temperature values and associated uncertainties have been established for widespread use in both metrology and industrial practice [18,19]. These temperatures are defined via the point of inflection (POI) and the liquidus point (LP), as described in Section 4. Concurrently, considerable effort has been invested in developing methodologies that support the practical, robust, and traceable realization of fixed-point-based temperature scale implementation.

It is worth noting that, within the ITS-90, the phase transition temperature of at least one of the three pure-metal fixed points—the freezing points of silver (Ag, *T*_90_ = 1234.93 K), gold (Au, *T*_90_ = 1337.33 K), and copper (Cu, *T*_90_ = 1357.77 K)—is widely employed by laboratories as a reference for establishing the radiation temperature scale above the silver point. However, this approach requires precise knowledge of the radiometer’s relative spectral response, traceable to absolute measurements, and depends on extrapolation procedures to extend the scale to higher temperatures [1,20]. Consequently, due to the intrinsic uncertainties associated with fixed-point realization and the rules governing uncertainty propagation, the resulting uncertainties increase substantially, reaching several kelvin near 3500 K, as discussed in Section 5. A further limitation has been the absence of robust fixed points above the copper freezing point. At higher temperatures, additional technical challenges arise, primarily due to interactions between metals and cavity materials—most commonly graphite—which render pure metals with higher melting or freezing points impractical for fixed-point realization.

In contrast, the development of metal–carbon eutectic and metal–carbide–carbon peritectic fixed points have created new opportunities for establishing the thermodynamic temperature scale with substantially reduced uncertainty [21,22,23,24,25]. Through the coordinated efforts of the aforementioned community, seven reference temperatures—well distributed across the high-temperature range—have been established, together with their associated uncertainties, covering temperatures from approximately 1200 K up to 3500 K [26,27,28]. Table 1 summarizes the current status of these reference temperature values and their assigned uncertainties [18,19].

The availability of these reference thermodynamic temperatures has enabled the practical implementation of RPRT for high-temperature thermodynamic measurements. This approach is based on relative radiometric measurements between reference temperatures and does not require prior knowledge of the spectral responsivity of the radiometer depending on the number of the used reference points [29,30], as described in Section 2. Among the primary thermometry methods described in the *MeP*-K, it is considered the most straightforward to implement, as it can be realized using various numbers and combinations of fixed points in conjunction with an interpolation pyrometer [31]. This methodology represents one of the central objectives of the ongoing EU-funded research project MultiFixRad [32], which aims to disseminate RPRT capabilities for the realization and dissemination of the kelvin at high temperatures across Europe [33,34].

However, despite its practical simplicity, realization of thermodynamic temperature via RPRT relies on a mathematically rigorous measurement framework [35,36,37,38,39]. The overall uncertainty is obtained by combining the uncertainties associated with the measurement process and the in-use uncertainty of the radiometer with the uncertainty contribution arising from the physical interpolation model [35]. The latter depends on the number and distribution of the fixed points employed and is evaluated through formal mathematical uncertainty propagation in accordance with the GUM framework [40].

In this work, a practical implementation of the RPRT method, together with a dedicated software package for temperature scale realization and uncertainty evaluation, is presented. The software, named MultiFixRadSoft, is an open-source package for RPRT realization developed in accordance with the *MeP*-K. It enables the realization of the thermodynamic temperature scale using the ITS-90 fixed points and a series of metal–carbon and metal–carbide–carbon eutectic and peritectic fixed points, for both narrow-band radiation thermometers and radiometers. Routines for the realization of ITS-90 above the silver point are also incorporated. In addition, the software provides routines for analyzing eutectic melting plateaux, enabling the determination of the point of inflection, liquidus point, and the melting range of the fixed-point realization. Furthermore, software provides correction methods for the size-of-source effect (SSE), detector nonlinearity, emissivity, and temperature drop.

The implementation and validation of the software tool are demonstrated through the practical realization of RPRT using six points (Cu, Fe–C, Co–C, Pd–C, Ru–C and WC–C) in conjunction with a linear radiation thermometer. Furthermore, the characterization of the radiometer within the relative primary radiometry framework, as well as its application as an interpolation device, are described.

MultiFixRadSoft provides a novel open-source and modular alternative to existing tools, significantly improving transparency, flexibility, and accessibility for relative primary radiation thermometry. It uniquely integrates realization of temperature scale, corrections, traceability, and rigorous uncertainty evaluation within a single platform, addressing a key gap in current software solutions. Unlike existing tools, it is readily adaptable by emerging NMIs/DIs, reducing barriers to adoption under the *MeP*-K framework. Additionally, it enhances reproducibility and supports training and knowledge transfer, facilitating wider implementation in both metrology and industry as well as future seamless integration into Digital Calibration Certificates (DCC) workflows, enabling traceability, interoperability, and compliance with modern digital metrology practices. MultiFixRadSoft v1.0.0 is released under a Clear BSD license via GitHub (v3.5.5) and the Python Package Index (PyPI—pip v23.3.1) and the MATLAB code, with full documentation hosted on GitHub.

The software is compatible with MATLAB (R2020a+) and Python (3.8+). The Python version requires NumPy (2.3.5), SciPy (1.10.0), and Matplotlib (3.7.0), while the MATLAB version uses standard functions and common toolboxes. All dependencies are documented to ensure reproducibility and consistent performance across both platforms.

The remainder of this paper is organized as follows. Section 2 and Section 3 outlines the means for realization of RPRT at UME. Section 4 describes software capabilities for the thermodynamic scale realization routines including the ITS-90 approach. Section 5 presents the software capabilities for uncertainty calculations. The validation of the software, case study and conclusions are provided in Section 6, Section 7 and Section 8, respectively.

## 2. Implementing Relative Primary Radiometry in Practice

**Figure 1:**
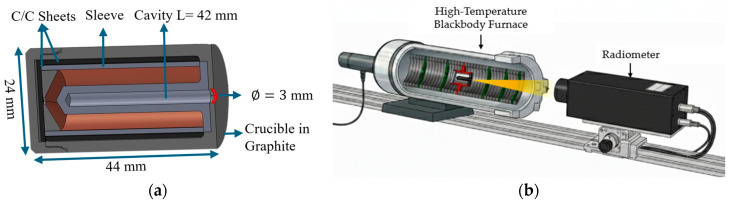
Schematic representation of: (**a**) the experimental setup; and (**b**) the HTFP cell.

In this section, before presenting the structure, capabilities, and mathematical foundations of the MultiFixRadSoft software package, the experimental setup employed for implementation of the RPRT method using high-temperature fixed points (HTFPs) is described. Figure 1 illustrates the experimental setup for fixed-point realization, comprising a high-temperature blackbody employed for the realization of fixed-point cells and a filter radiometer. A brief description of each component is provided below.

### 2.1. HTFP Cells

In the present study, the six HTFPs were realized. The fixed point with the lowest phase transition temperature is a copper point cell (Cu cell) containing high-purity metal. For the scale realization, only this cell was used in the freezing mode, and the corresponding freezing signal was measured. For all remaining cells—including five metal–carbon eutectic fixed points (namely, Fe–C, Co–C, Pd–C, and Ru–C) as well as one metal–carbide–carbon peritectic fixed point (WC–C)—the melting signals were used for the interpolation routines and temperature scale realization. All cells were manufactured according to standardized designs that ensure robustness and long operational lifetime, resulting from extensive collaborative efforts within the metrology community over the past three decades. The cavities of the fixed-point blackbodies were made of high-density graphite, with a clear aperture of 3 mm and a cavity length of 42 mm, yielding an emissivity greater than 0.9997. Figure 1a presents a schematic of the cell design. The cells employed in this study were developed at UME within the framework of the Real-K [41] and MultiFixRad projects.

### 2.2. High Temperature Furnace

All cells were realized in an HTBB 3500M (VNIIOFI, Moscow, Russia) high-temperature furnace, as shown in Figure 1b. In this furnace, a series of pyrolytic graphite rings, compressed by a spring between the front and rear electrodes, form a resistance-type cylindrical heating tube. By positioning rings with higher electrical resistivity near the ends of the heating tube to compensate for end heat losses, and rings with nearly uniform resistivity in the central region, the temperature uniformity in the central part of the furnace is effectively controlled [42]. Temperature uniformity within the tube was further enhanced through the use of five baffles: two positioned behind the cell holder and three placed in front of the cell. The front baffles have different aperture diameters to avoid vignetting effects. To fix the HTFPs inside the furnace, a cell holder made of high-density graphite is used. Pieces of carbon–carbon (C/C sheet) composite sheet are employed to center the cell within the holder.

### 2.3. The Radiometer

**Figure 2:**
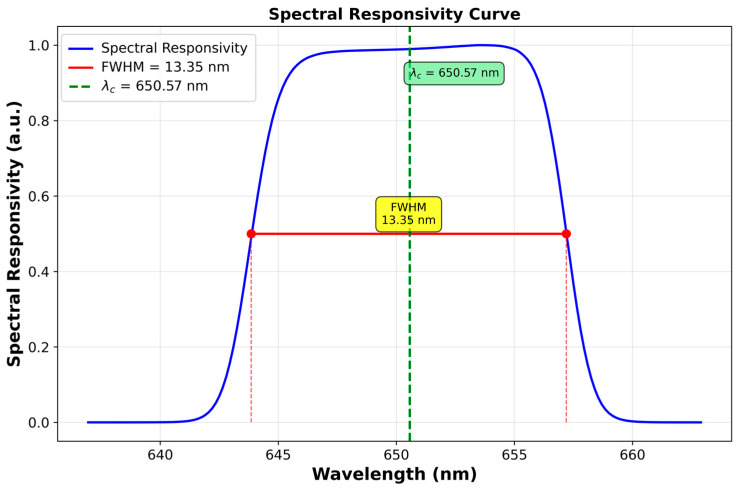
Normalized spectral responsivity of the LP5.

The radiometer used in this study is an LP5 linear pyrometer (IKE, Stuttgart, Germany) operating as a radiation thermometer with a central wavelength of 650 nm. It is equipped with an optical bandpass filter having a full width at half maximum (FWHM) bandwidth of 13.35 nm, corresponding to a relative bandwidth of $\frac{\sigma }{\lambda }=0.0061$. This results in a negligible uncertainty contribution to temperature even at 3500 K [43]. Figure 2 shows the relative absolute spectral responsivity of the LP5, which is traceable to a cryogenic radiometer. The LP5 has a spot size of 0.7 mm at an 800 mm focal plane. The instrument was mounted on three-axis precision translation stages and two tilt-adjustment mechanisms to accurately align the pyrometer with the cavity aperture. In the following section, the characterization of the radiometer in the context of relative primary radiometry is described.

### 2.4. Characterization of the LP5

#### 2.4.1. Size-of-Source (SSE)

The SSE of a radiometer is a parameter that characterizes the contribution of radiation from outside the nominal source area to the detected signal [44]. The SSE of the LP5 was determined using the indirect method. A 500 mm diameter integrating sphere with a 100 mm clear output aperture was used as the radiation source. The sphere was illuminated by four incandescent lamps, with internal baffles placed in front of the lamps to ensure a uniform irradiance distribution at the exit port.

A black graphite spot with a diameter of 3 mm, fixed at the center of a quartz window, was used as an obscuration. A second quartz window without an obscuration was used to measure and subtract the background signal. Diaphragms with different aperture diameters were employed to vary the effective source size.

**Figure 3:**
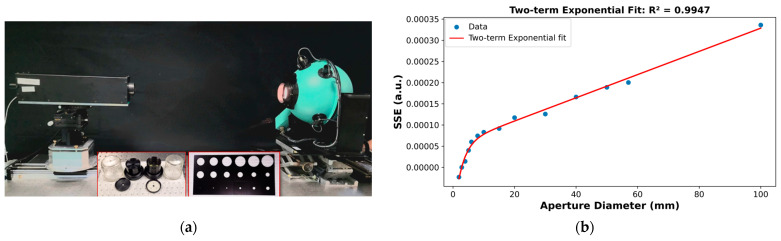
(**a**) The SSE measurement setup; (**b**) The SSE measurement and fit results for the LP5.

Figure 3a shows the experimental setup, while Figure 3b presents the results of the SSE characterization of LP5. These results were used to correct the LP5 output signal for SSE, accounting for radiation originating outside the nominal cavity aperture, as described in Section 4.5.1.

#### 2.4.2. The Radiometer Linearity

Linearity of a radiometer is defined as the degree to which its output signal is directly proportional to the incident radiant power over its operating range. The LP5 radiometer is generally characterized by excellent linearity; nevertheless, its linearity was explicitly assessed in this work. For temperatures from Cu-point to 1900 K, a flux-doubling method employing two high-stability lamps (lamp-based radiance method) was used. At higher temperatures, a double-aperture method was applied. The resulting data were used to correct the LP5 output signal with an uncertainty value described in Section 5. The software performs a linear fit to the tabulated linearity data and applies the corresponding correction to the output signal, as described in Section 4.5.2.

### 2.5. Determination of Thermodynamic Temperature by the RPRT Method

As mentioned previously, within spectral-band radiometric thermometry, RPRT is formally recognized as a primary method in the *MeP*-K, providing a practical alternative to absolute radiometry for the realization of high-temperature scales.

The mathematical foundation of RPRT is based on the Planck-form Sakuma–Hattori equation [45]:

(2) $$S\left(T\right)=\frac{C}{\exp\left(\frac{c_{2}}{AT+B}\right)-1}$$

which expresses the radiometer signal *S(T)* as a function of thermodynamic temperature *T* through a set of fit coefficients *A*, *B*, *C* and the second radiation constant $c_{2}=hc/k_{B}$. Provided the spectral band is narrow [35],

(3) $$A=\lambda_{0}\left(1-6\frac{\sigma^{2}}{\lambda_{0}^{2}}\right),\;B=\frac{c_{2}}{2}\frac{\sigma^{2}}{\lambda_{0}^{2}}$$

The mean wavelength of the relative spectral responsivity $s\left(\lambda \right)$ is defined as

(4) $$\lambda_{0}=\frac{\int_{0}^{\infty }\lambda s\left(\lambda \right)d\lambda }{\int_{0}^{\infty }s\left(\lambda \right)d\lambda }$$

and the variance (related to the bandwidth) is

(5) $$\sigma^{2}=\frac{\int_{0}^{\infty }{\left(\lambda -\lambda_{0}\right)}^{2}s\left(\lambda \right)d\lambda }{\int_{0}^{\infty }s\left(\lambda \right)d\lambda }$$

Both quantities are determined from spectral responsivity measurements of the radiometer. For spectral responsivity profiles of approximately rectangular shape, such as that of LP5, the bandwidth parameter can be approximated by $\sigma \approx \frac{FWHM}{2\sqrt{3}}$ [35].

**Figure 4:**
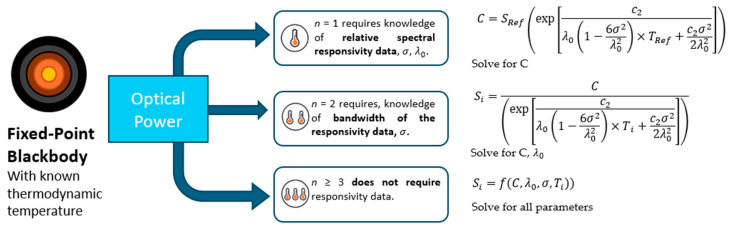
Relative primary thermodynamic temperature scale realization routes.

RPRT realization inputs and outputs are summarized in Figure 4. There are now three recognized schemes to determine the constants in Equation (2). For the *n* = 1 scheme, the parameters A and B are determined from Equations (3)–(5), while the parameter C is calculated from Equation (6) using the reference signal $S_{ref}$ corresponding to the known reference temperature $T_{ref}$:

(6) $$C=S_{Ref}\left(\exp\left[\frac{c_{2}}{\lambda_{0}\left(1-\frac{6\sigma^{2}}{\lambda_{0}^{2}}\right)\times T_{Ref}+\frac{c_{2}\sigma^{2}}{2\lambda_{0}^{2}}}\right]\right)$$

For the *n* = 2 scheme, two reference signals, $S_{ref,\;1}\;$ and $S_{ref,\;2}\;$ corresponding to the reference temperatures $T_{ref,\;1}\;$ and $T_{ref,\;2}\;$ are used. The two resulting equations are applied to determine the unknown parameters C and $\lambda_{0}$ according to Equation (7), with *i* = 1 and 2:

(7) $$S_{ref,i}=\frac{C}{\left(\exp\left[\frac{c_{2}}{\lambda_{0}\left(1-\frac{6\sigma^{2}}{\lambda_{0}^{2}}\right)\times T_{ref,\;i}+\frac{c_{2}\sigma^{2}}{2\lambda_{0}^{2}}}\right]\right)}$$

For the *n* ≥ 3 scheme, Equation (2) is solved without prior knowledge of the spectral responsivity parameters (i.e., *σ* and _0_), since the number of reference equations is equal to or greater than the number of unknown fit coefficients.

### 2.6. Software Workflow Diagram

The software, developed in accordance with the RPRT procedure, provides a structured environment for processing data from HTFP melting plateau realizations. The signal at the phase transition is extracted using two approved methods—namely, the POI and the LP—and melting range analysis is supported. At this stage, the interpolation routine given in Equation (2) may be applied, and a preliminary comparison between the calculated calibration temperature(s) and the reference values listed in Table 1 is performed to assess the suitability of the results.

**Figure 5:**
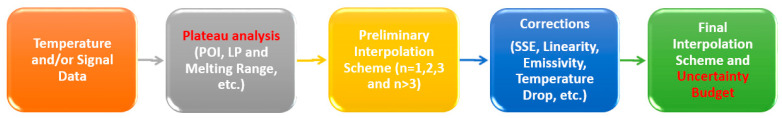
Flowchart of the software.

Subsequent processing includes corrections to the acquired signals for SSE, radiometer non-linearity, cavity emissivity, and cavity temperature drop effects. The corrected fixed-point signals are then used to establish the scale interpolation equation, and the associated uncertainty contributions are evaluated. The overall workflow of MultiFixRadSoft is shown in Figure 5.

Using the corrected data, an interpolation scheme based on the Sakuma–Hattori equation is generated, allowing calibration with different interpolation orders (*n* = 1, 2, 3, or *n* > 3), depending on the number of realized fixed points. Finally, the software produces a customizable uncertainty budget in accordance with [40]. While several predefined uncertainty components are provided, users are required to supply their own measurement and modeling contributions. Together with additional components recommended in [46], this enables a complete and traceable realization of the thermodynamic temperature scale consistent with the *MeP*-K.

## 3. Experiments

### 3.1. Plateau Realization

The fixed-point realization campaign was carried out from the highest to the lowest temperature; i.e., from the WC–C fixed point down to the Cu fixed point. The HTFP cells were positioned in the most temperature-uniform region of the furnace, as identified during the measurement campaign of the Real-K project. It is worth noting that, before commencing measurements with the subsequent HTFP, the furnace tube without a cell, together with the empty cell holder and the C/C-sheet pieces, were baked at approximately 2850 °C for about 1.5 h in order to eliminate possible metal contamination originating from the preceding cell.

**Figure 6:**
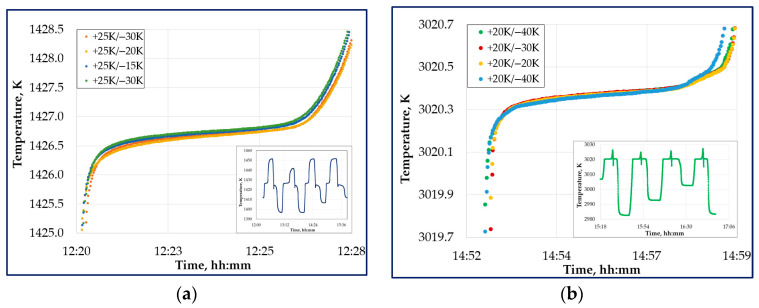
Typical melting curves for plateau realizations: (**a**) Fe–C cell; (**b**) WC–C cell. The upper-left insets show the furnace set values, while the lower-right insets illustrate the complete plateau realization process.

For each HTFP, four complete melting–freezing cycles were performed sequentially during a single furnace run. For several eutectic alloys, the melting temperature of the fixed-point cell is known to depend on the thermal history of the ingot in previous realizations [47,48]. Therefore, the measurement protocols with different melt steps (+15 K, +20 K, +25 K, and +30 K) and freeze steps (−15 K, −20 K, −30 K, and −40 K) were applied. The first melting–freezing cycle was excluded from the data analysis. The second, third, and fourth plateaus were used for the plateau analysis to evaluate the reference signal and associated uncertainties. Figure 6a,b show examples of plateaus obtained according to the measurement protocols.

### 3.2. The Radiance Temperature Distribution Across the Furnace

**Figure 7:**
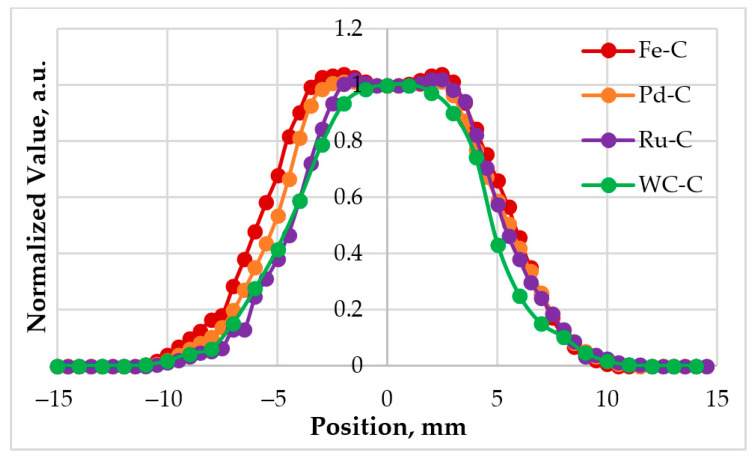
Normalized radiance temperature distributions measured across the furnace cavity aperture for HTFP realizations.

During the fifth realization, the radiance temperature distribution across the furnace aperture was retrieved by horizontally scanning the LP5 radiometer across the cavity with a computer-controlled linear translation stage. Figure 7 presents the normalized radiance temperature distributions measured during the melting phase transitions of the Fe–C, Pd–C, Ru–C, and WC–C HTFPs, while analogous measurements were also carried out at the Cu and Co–C fixed points. These data were subsequently used to apply the SSE correction to the radiometer output signals [49] during the HTFP melting transitions, as detailed in Section 4.5.1.

## 4. Software Implementation

In the following we illustrate the workflow in the software using the data acquired as described in the previous section, and explain in detail the analysis options implemented in each step. The software is open source and its architecture follows a modular design approach. Particular attention was paid during development to ensure flexibility and ease of modification. Depending on specific experimental requirements, users can readily implement their own methods for POI and LP evaluation methods, as well as corrections such as SSE, emissivity and nonlinearity.

### 4.1. Plateau Analysis and Data Preparation

**Figure 8:**
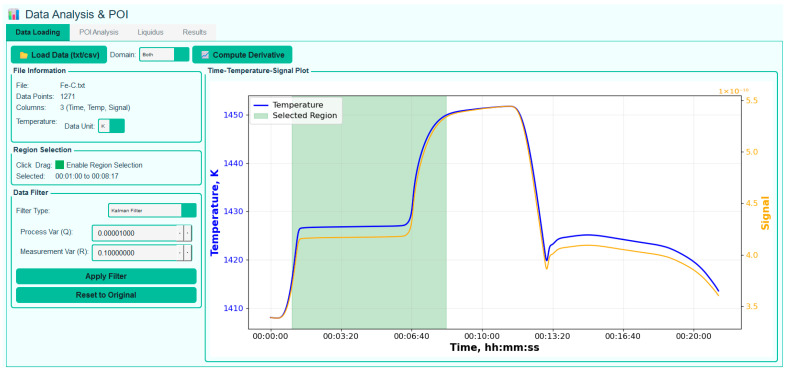
An example of the software user interface after loading the plateau data.

**Figure 9:**
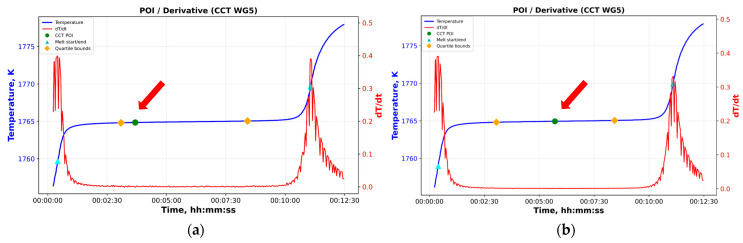
Impact of moving average filtering on the Pd-C HTFP melting curve POI; (**a**) noisy curve, (**b**) filtered curve showing ~0.1 K increase (red arrows point the POI values).

Following the establishment of the phase change plateaus, data analysis is initiated by importing the plateau data into the software through the graphical user interface (Figure 8). In the absence of reference temperature values, the temperature column may be set to zero. For data pre-processing, one of three filtering techniques—Kalman, Savitzky–Golay, or moving-average filtering—may be applied, and a region of interest on the plateau curve is selected. An example illustrating the impact of noise filtering on the determination of the POI is presented in Figure 9. Figure 9a shows a noisy melting curve of the Pd-C HTFP realization within the scope of the Real-K project. In contrast, Figure 9b presents the same melting curve after applying a moving average filter prior to POI evaluation. The filtering results in an increase of approximately 0.1 K in the POI value, which was subsequently confirmed by realizing the same fixed point with improved melting curve quality. Finally, the user defines the processing domain (temperature or signal) and selects the methods for POI and LP determination, as described below.

### 4.2. Point of Inflection (POI)

**Figure 10:**
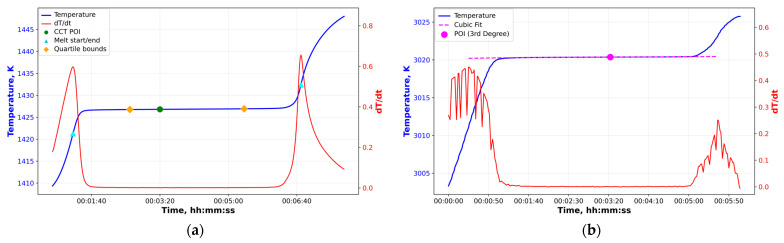
POI calculations; (**a**) the derivative method for the Fe–C HTFP cell and (**b**) the third-order polynomial fit (shown in pink line) method for the WC–C HTFP cell.

Except for the Cu fixed point, for which the phase transition temperature is determined from the freezing plateau using an analysis consistent with the ITS-90 procedures (the software allows flexible selection of the region of interest on the freezing curve and computes statistical parameters such as the mean, standard deviation, and minimum and maximum values), the melting curves of all eutectic HTFPs are analyzed by determining the POI. To this end, the software tool incorporates three alternative algorithms. The first method, recommended by CCT Working Group 5 and designated CCT-WG5-WP2 [50], smooths the melting plateau using a moving-average filter with an initial averaging window; this window is subsequently halved and doubled. The final POI is obtained as the mean of the three resulting values. Figure 10a illustrates an example application of this method for POI determination using the melting curve of the Fe-C HTFP.

The second method, third-order polynomial fitting [18], fits a cubic function, given by Equation (8), to the central half of the plateau. The POI temperature (or corresponding signal) is then calculated by substituting $t=-\frac{b}{3a}$ into Equation (8).

(8) $$T=at^{3}+bt^{2}+ct+d$$

Figure 10b illustrates an example application of the third-order polynomial fitting method for POI determination using the melting curve of the WC–C HTFP. The POI values that are used in this work summarized in Table 2.

The third method, the selective multiple-fit approach [51] applies repeated third-order polynomial fits over variable intervals defined by extrema of the first and second derivatives; only statistically consistent fits are averaged to determine the final POI. As this method is primarily advantageous for fixed-point measurements with large cavity sizes, it was not applied in the present work, although it is implemented in the software.

### 4.3. Liquidus Point (LP)

**Figure 11:**
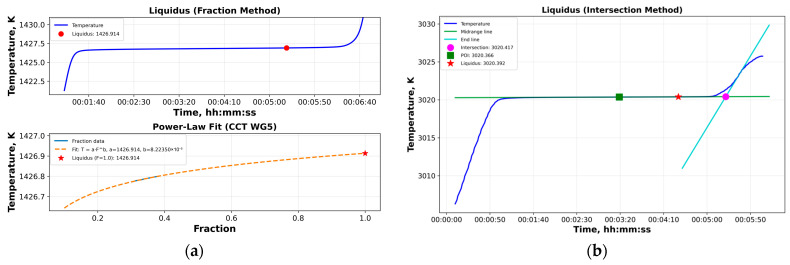
LP calculations using (**a**) the fraction method for the Fe–C HTFP cell and (**b**) the intersection method for the WC–C HTFP cell.

Analogous to the determination of the POI, the LP may be determined using two established approaches; namely, the fraction method and the intersection method, which are both commonly applied. The developed software tool incorporates both methods. In the fraction method [48], the melt fraction *F* is derived under the assumption of a constant rate of heat input. Equation (9) is fitted to the data from the second quartile of the melting plateau up to the POI and is subsequently extrapolated to F=1  to estimate the liquidus temperature (or corresponding signal), as shown in Figure 11a.

(9) $$T\left(F\right)=T_{liq}F^{a}$$

The intersection method [47] employs two linear fits: one to the data in the vicinity of the POI and another to the post-melting region, where the derivative of the signal reaches its maximum. The intersection of these two lines defines the upper liquidus limit as shown in Figure 11b.

The equilibrium liquidus temperature (or signal) is then taken as the midpoint between the POI and this upper limit, assuming a rectangular probability distribution. The melting range is calculated automatically as the product of the slope of the tangent at the POI and the duration of the melting plateau. Figure 11a,b depict the implementation of both methods for determining the LP values from the melting plateaus of Fe–C and WC–C, respectively, and Table 3 summarizes the numerical data.

### 4.4. Preliminary Scale Realization

**Figure 12:**
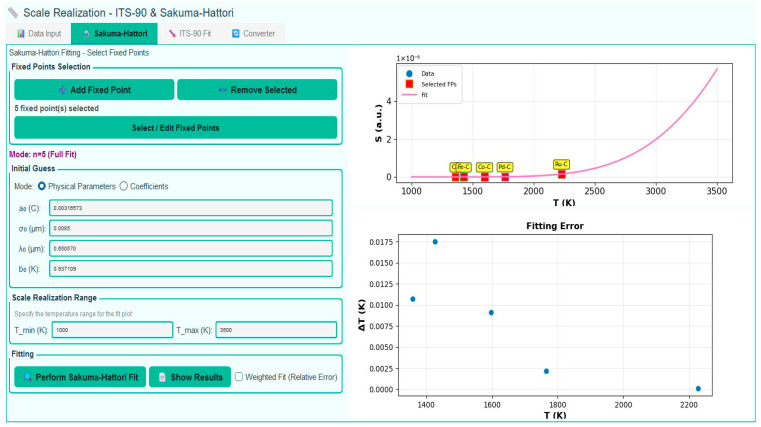
Preliminary scale realization user interface, n = 5 scheme.

This feature is provided as an optional tool within the software, intended primarily for ad hoc evaluations during the initial realization stage of each fixed point. At this preliminary phase—before any corrections are applied and prior to final scale realization—it is considered good practice to verify the internal consistency of the measured signals. This can be achieved by fitting the data to the Sakuma–Hattori Equation (2) or to ITS-90 routines, as illustrated in Figure 12, thereby enabling the retrieval of temperature values with at least approximate accuracy. The resulting fit expresses signal as a function of temperature through a set of fitted coefficients *A*, *B*, and *C*. Examination of these coefficients, together with the residuals, provides an early diagnostic of the internal consistency of both the HTFP realizations and the plateau analysis.

### 4.5. Corrections

The MultiFixRadSoft software incorporates built-in modules to apply corrections for the principal sources of systematic error, including the SSE, detector linearity, emissivity, and temperature drop. While SSE and linearity affect the apparent radiance measured by the radiation thermometer, emissivity and temperature drop correspond to physical effects that reduce the actual radiance emitted by the source.

#### 4.5.1. SSE Correction

As mentioned above, during the measurements the radiometer detects not only radiation emitted from the cavity aperture but also unwanted contributions from stray radiation originating outside the aperture, including furnace emission and interreflections. The radiometer signal is therefore corrected for these effects, referred to as the SSE correction, prior to final realization of the scale, using the radiometer SSE curve (Figure 3b) together with the measured radiance temperature distribution across the furnace aperture (Figure 7).

The SSE contribution can be treated as a convolution of the radiometer SSE function with the radiance temperature distribution across the furnace aperture. Several methods exist to recover the pure cavity signal and apply the corresponding correction. Depending on the shape of the radiometer SSE curve, the software fits one of several models—polynomial, exponential, logarithmic, power-law, or two-term exponential—to describe the SSE behavior; alternatively, it allows the user to implement a custom equation. Using the fitted SSE model, relevant geometrical parameters, including the nominal target size (3 mm in the present work), the cavity clear aperture, the furnace aperture size, and the radiance temperature distribution across the furnace aperture, the SSE correction is automatically calculated by the software.

Based on these inputs, the measured signal is adjusted according to the geometric relationship between the target and the blackbody aperture. If the target size exceeds the cavity aperture, the corresponding contribution is added to the signal; if it is smaller than or equal to the aperture, the contribution is subtracted, as expressed in Equation (10), where $S_{c}$ is the corrected signal and $S_{m}$ is the measured signal [52].

(10) $$S_{c}=\left\{\begin{array}{c} \left(1+\frac{\Delta L}{L_{\lambda }}\right)\cdot S_{m},if\;Target>Aperture\;size\\ \left(1-\frac{\Delta L}{L_{\lambda }}\right)\cdot S_{m},\;if\;Target\;\leq \;Aperture\;size \end{array}\right.$$

In the present setup, the SSE contribution is positive and is therefore subtracted from the measured radiometer signal to retrieve the true cavity radiance signal. The implementation utilizes a ring-by-ring integration methodology for SSE correction.

#### 4.5.2. Non- Linearity Correction

The non-linearity correction factors were calculated by first normalizing the correction at the lowest $S_{1+2}$ signal. Subsequently, for each higher $S_{1+2}$ signal, the corresponding correction factor was obtained by cumulatively multiplying all preceding linearity values [53]. Intermediate correction factors were derived by performing a linear interpolation between the two nearest points surrounding the given signal value. This procedure produces a continuous correction function C(I), which can be applied to measured signals to compensate for cumulative detector non-linearity effects.

#### 4.5.3. Emissivity Correction

An additional correction accounts for the finite emissivity of the HTFP cavity, which is lower than that of an ideal blackbody. The emissivity correction for the cavities of the HTFP cells investigated in this work was calculated using Equation (11):

(11) $$∆T=\left(1-\varepsilon \right)\left(\lambda_{0}T^{2}/c_{2}\right)$$

where $\lambda_{0}$ is the central wavelength of the radiation thermometer and *T* is the measured temperature. All cells have comparable cavity geometries and employ high-purity, high-density graphite as the wall material. For all HTFP cells, an isothermal cavity emissivity of *ε* = 0.9997 was assumed [54].

#### 4.5.4. Temperature Drop Correction

In HTFPs, a small temperature gradient may develop across the cavity back wall due to thermal conduction and radiative losses through the aperture. This effect is referred to as the temperature drop. An approximate model to describe it assumes that the heat loss from the surface occurs via the direct line of sight from the cavity bottom, through the cavity aperture, in to ambient. For a cylindrical cavity with a conical bottom, the temperature drop can be estimated as Equation (12):

(12) $$\Delta T_{drop}=\left(cos\theta \cdot \varepsilon_{\lambda }\cdot \sigma \cdot \frac{k}{d}\right).{\left(\frac{r}{L}\right)}^{2}\cdot T_{base}^{4}$$

Here, θ is the tilt angle of the cavity bottom, $\varepsilon_{\lambda }$ is the emissivity of graphite at the working wavelength of the radiometer, σ is the Stefan–Boltzmann constant, k is the effective thermal conductivity of the cavity material, d is the cavity wall thickness, L is the cavity length, r is the cavity radius, and $T_{base}\;$ is the measured base temperature of the cavity [55]. Equation (12) is a worst case for the temperature drop, because the side wall of the cavities will partly make up for the heat loss through the aperture. Default values for these parameters corresponding to the cells used here are provided in the software and may be adjusted by the user as required. Using Wien’s approximation, this temperature difference is also expressed as a correction to the measured radiometer signal.

Table 4 summarizes the corrections applied to all HTFP signals used in this work.

### 4.6. Scale Realization

**Figure 13:**
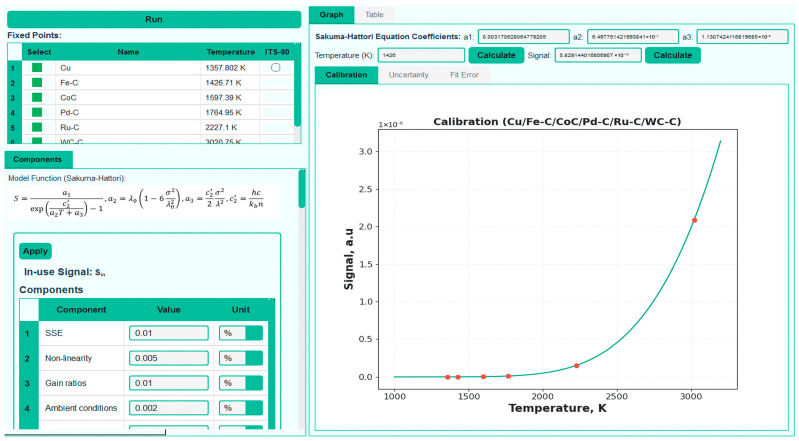
Thermodynamic temperature scale for the n = 6 scheme.

Finally, after applying the signal correction procedures, the final scale realization is performed using the corrected signals corresponding to each available HTFP. Depending on the number of available corrected fixed-point signals, different interpolation schemes can be applied, corresponding to cases with n=1,n=2, n=3 or n>3. As discussed in the previous section, the Sakuma–Hattori equation contains three unknown fitting coefficients. Consequently, for scale realizations based on n=1 or n=2, prior knowledge of the relative spectral responsivity of the radiometer is required. For cases where n≥3, the number of equations is equal to or greater than the number of unknowns, allowing the interpolation fitting to be performed without requiring any spectral information about the radiometer. The MultiFixRadSoft software automatically selects the appropriate interpolation scheme based on the number of selected reference data. Figure 13 illustrates the realization of the thermodynamic temperature scale using the HTFPs employed in this work.

In addition, the software incorporates complete routines for ITS-90 scale realization using extrapolation equations, as described in the next section, provided that the corrected signal at the copper (Cu) fixed point—or, alternatively, at the silver (Ag) or gold (Au) fixed points—and the spectral responsivity of the radiometer are available. It is worth noting that similar extrapolation routines are also available for any HTFP above the Cu point, as described in the following subsection.

### 4.7. ITS-90 Routines

The spectral responsivity of the LP5 was measured using a double monochromator (DTMc300, Bentham Instruments Ltd., Reading, UK). As shown in Figure 2, the normalized spectral responsivity was determined by referencing the measurements to a silicon photodiode with spectral responsivity traceable to a cryogenic radiometer.

The software tool performs the ITS-90 scale realization once the relative spectral responsivity, a reference signal from at least one ITS-90 metal fixed point (e.g., Ag, Au, or Cu), and the required experimental data and correction terms are provided. Commonly, above the silver point, temperatures on ITS-90, denoted by$\;T_{90}$, are defined by Equation (13) [56]:

(13) $$\frac{L_{\lambda }({\;T}_{90})}{L_{\lambda }({\;T}_{90,X})}=\frac{\exp\left(\frac{c_{2,90}}{{\;\lambda T}_{90,X}}\right)-1}{\exp\left(\frac{c_{2,90}}{{\;\lambda T}_{90}}\right)-1}$$

where ${\;T}_{90,X}$ refers to one of the silver (${\;T}_{90,Ag}=1234.93\;K)$, gold ($T_{90,Au}=1337.33\;K)$, or copper ($T_{90,Cu}=1357.77\;K)$ freezing points; $L_{\lambda }({\;T}_{90})$ and $L_{\lambda }\left({\;T}_{90,X}\right)\;$are the spectral concentrations of the radiance of a blackbody at the wavelength (in vacuum) λ at ${\;T}_{90}\;$ and at ${\;T}_{90,X},\;$respectively; and $c_{2,90}\;$is the second radiation constant, defined on ITS-90 as $c_{2,90}=0.014388\;m\cdot K$.

In addition, the software allows implementation of the extrapolation approach defined in Equation (14), in accordance with the ITS-90 procedure, for arbitrary HTFPs. The extrapolation is performed by applying the Newton–Raphson iterative method using Equation (13), as formulated in Equation (14). For the n = 1 scheme, the ITS-90 reference temperature $T_{90}$ and the corresponding reference signal $S_{ref}$ in Equation (14) are replaced by the selected HTFP reference temperature and its corresponding reference signal.

(14) $$T_{90,i+1}=T_{90,i}+\frac{\int_{0}^{\infty }L_{\lambda }\left(T_{90,ref}\right)s\left(\lambda \right)d\lambda \times \frac{S_{meas}}{S_{ref}}-\int_{0}^{\infty }L_{\lambda }\left(T_{90,i}\right)s\left(\lambda \right)d\lambda }{\frac{c_{2}}{T_{90,i}^{2}}\int_{0}^{\infty }\frac{L_{\lambda }\left(T_{90,i}\right)s\left(\lambda \right)}{\lambda \left[1-exp\left(-\frac{c_{2}}{\lambda T_{90,i}}\right)\right]}d\lambda }$$

It is worth noting that the tool also performs the uncertainty analysis for the ITS-90 realization and evaluates the extrapolation uncertainty in accordance with the law of propagation of uncertainty. For the case n = 1, scale realization and uncertainty evaluation based on any HTFP follow a procedure analogous to that of the ITS-90; however, in this mode, uncertainties associated with the thermodynamic realization of the fixed point itself are required.

## 5. Uncertainty Evaluation

As noted above, the core functionality of the MultiFixRadSoft tool is the comprehensive uncertainty analysis for RPRT-based scale realization. The mathematical framework of this analysis is mainly based on the work of Saunders et al. [37] and on [57]. In version 2 of the software, some minor misprints identified in the equations reported in [37] were corrected in accordance with [57].

A key feature of the tool is its flexibility in uncertainty evaluation. Individual uncertainty components may be included or excluded via the user interface, according to the specific experimental setup and measurement procedure, in addition to those prescribed by the standard RPRT methodology, without modification of the underlying code. This flexibility is particularly important during scale realization, where experimental configurations and underlying assumptions may differ.

In general, the uncertainty budget in an RPRT-based scale realization comprises several main categories, including contributions from interpolation routines based on the physical interpolation equations, the in-use uncertainty of the radiometer, uncertainties associated with the realization of the HTFPs, and uncertainties related to applied correction terms. The uncertainty budget for each HTFP used in this study for the practical implementation of the RPRT is presented in Table 5.

Based on the measured and corrected data, a fit using the Sakuma–Hattori interpolation is performed and a comprehensive uncertainty budget is generated automatically, including fit residuals and the measurement-related uncertainty components mentioned above. This ensures that the resulting temperature scale is traceable to thermodynamic principles in accordance with the *MeP*-K.

In radiation thermometry, the evaluation of temperature uncertainty within the Sakuma–Hattori model requires the propagation of multiple, often correlated quantities, such as the effective wavelength, bandwidth, reference temperature, and calibration signals. Traditionally, this requires explicit calculation of sensitivity coefficients and uncertainty propagation for n = 1, 2, 3, and n > 3 realizations, which is mathematically intensive and prone to manual error. MultiFixRadSoft automates this process by performing all partial derivative and uncertainty propagation calculations internally.

For n = 1 and n = 2 realizations (as well as for ITS-90 routines), the software is used to compute the effective wavelength and bandwidth parameters required for evaluation of the Sakuma–Hattori equation. Uncertainty components related to the spectral responsivity—such as wavelength alignment, filter transmittance, and detector responsivity—must be determined independently by the user prior to the analysis. These contributions are incorporated into the overall uncertainty budget by the software but are not estimated automatically.

**Figure 14:**
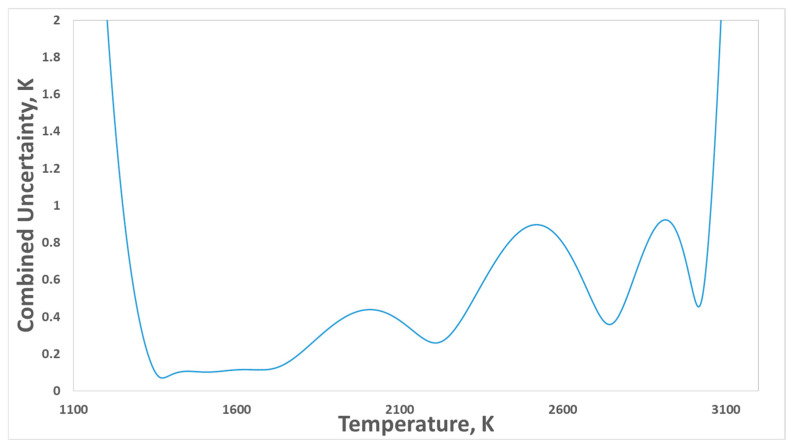
Combined uncertainty analysis of a PRRT scale established using all HTPFs employed in this study (*n* = 7 scheme).

In contrast, for n = 3 and n > 3 realizations, relative spectral responsivity data are no longer required. In this case, the software performs a multiple fixed-point interpolation directly using the corrected signals and the corresponding thermodynamic temperatures. Each realization requires the signal and temperature uncertainty components associated with each fixed point. Figure 14 presents the combined uncertainty generated by the software from the experimental data obtained during the practical implementation of the RPRT. In overdetermined systems (n > 3) where the calibration equation is determined via the least-squares method, the sensitivity coefficients—which quantify how uncertainties in the calibration data propagate to the dependent variable—are analytically derived by evaluating the partial derivatives of the minimized χ^2^ objective function [58]. Unlike exact-fit scenarios where coefficients are found directly, least-squares parameters depend collectively on the entire calibration dataset; therefore, their sensitivities must be extracted from the optimization condition itself. This analytical derivation allows the impact of individual calibration uncertainties on the interpolated results to be systematically evaluated. For n > 3, uncertainty is calculated according to [58].

Table 5 and Table 6 detail the uncertainty budgets for each HTPF used in this study for the practical implementation of the RPRT, as well as for ITS-90 scale realization at the Cu point.

Although the software provides a predefined set of standard uncertainty components for guidance, users may modify or extend these components to reflect their specific experimental configuration.

## 6. Validation of the Software

The verification, validation, and testing of the software were systematically performed throughout the entire software development life cycle. Each computational module—including SSE correction, linearity correction, emissivity correction, temperature-drop correction, and rigorous uncertainty evaluation—was independently verified through manual calculations and cross-comparison of results. The strong agreement between the software outputs and manually evaluated values confirms the correctness and robustness of the implemented algorithms. Furthermore, as noted previously, the temperature scale realization obtained using the software was found to be in full agreement with results derived from manual analysis conducted within the Real-K project, thereby providing additional confidence in the software’s reliability and accuracy.

In addition, it is important to emphasize that one of the primary objectives of the MutiFixRad project is to support the development of low-cost interpolation radiation thermometers and to facilitate the dissemination of radiation temperature scales based on such devices, particularly for emerging NMIs and DIs. As demonstrated by Lowe [31], such instruments can achieve uncertainties comparable to primary standards, with traceability to the kelvin above 1300 K, in line with proposed revisions to the *MeP*-K.

Within this framework, UME upgraded its low-cost radiation thermometer and optimized it to operate as an interpolation device for realizing a radiation temperature scale above 1300 K with low uncertainty. The instrument incorporates a narrow-band interference filter centered at 900 nm, a temperature-stabilized silicon detector with a 100 mm^2^ active area, and a multi-stage gain-switching transimpedance amplifier. Its metrological characteristics were assessed through measurements of the gain ratio, linearity, Size-of-Source Effect (SSE), and sensitivity to ambient temperature variations. Calibration was performed using the copper fixed point together with several high-temperature fixed points (HTFPs); namely, the Fe–C, Co–C, Pd–C, Ru–C and WC–C eutectic cells. The radiation temperature scale was subsequently established using the MultiFixRadSoft tool.

The realized temperature scale was then compared with that obtained from a primary standard LP5 pyrometer using two blackbody furnaces: a variable-temperature blackbody with a cylindrical cavity and a 50 mm aperture covering the range 1200–1900 K, and the BB3500 blackbody furnace with a conical cavity and a 35 mm aperture covering 1500–2500 K. The comparison showed agreement between the two scales within the associated uncertainties. As reported in our previous work [59], these findings provide experimental validation of the MultiFixRadSoft tool and demonstrate its applicability to a broad range of instruments. The results further indicate that low-cost interpolation radiation thermometers can effectively support the realization and dissemination of the radiation temperature scale in accordance with the *MeP*-K.

## 7. Case Study: Optimization of the Calibration Scheme

**Figure 15:**
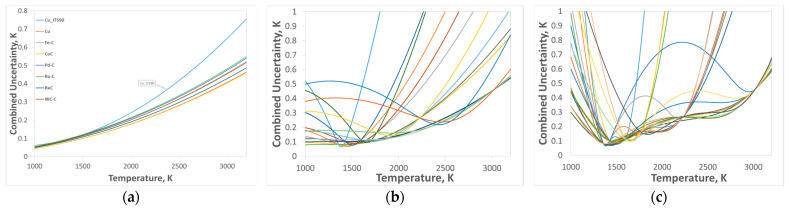
Representative uncertainty analysis plots obtained using the six HTFPs, (**a**) ITS-90 routines based on the Cu fixed point, and the n = 1 scheme; (**b**) n = 2 scheme (all possible combinations of two HTFPs); and (**c**) n = 3 scheme (all possible combinations of three HTFPs).

The realization of thermodynamic temperature scales using RPRT offers considerable flexibility in the choice of reference temperatures. This flexibility enables an optimization of the choice based on the temperature range to be covered by the radiometer, by seeking to minimize the uncertainty in the range of interest. However, over the full temperature range from approximately 1200 K to 3500 K, the overall uncertainty behavior is non-trivial due to the combined effects of interpolation and extrapolation inherent to the scale realization process. For schemes with n = 1, as well as for ITS-90-based realizations, only extrapolation equations (Equations (13) and (14)) are employed. Consequently, in accordance with the law of uncertainty propagation, the total uncertainty increases monotonically with increasing the temperature (Figure 15a).

For schemes with n ≥ 2, both interpolation and extrapolation are involved, each contributing distinct advantages and limitations. While increasing the number of HTFPs can reduce the uncertainty within the interpolated temperature range, the uncertainty associated with extrapolation beyond this range may increase significantly: Figure 14 shows an example for n = 7 where the uncertainty beyond the calibration range increases rapidly. In addition, the use of a larger number of HTFPs leads to increased practical complexity and cost, particularly for fixed points based on materials such as palladium, platinum, or rhenium, where achieving the required material purity is costly. Further challenges arise from the fabrication of HTFP cells, as some materials require advanced expertise to ensure reliable filling, stability, and operation.

In this context, when all HTFPs listed in Table 1 are considered, the total number of possible combinations becomes substantial. For the n = 2 scheme, there are 28 possible combinations, while for the n = 3 scheme, the number increases to 56. Performing the scale establishment—and, more critically, the associated uncertainty analysis—for each configuration manually is practically infeasible. Therefore, the automated routines provided by the MultiFixRad software are essential for systematic scale realization and rigorous uncertainty evaluation across all possible HTFP combinations. For example, Figure 15b illustrates the n = 2 scheme considering all possible combinations of the six HTFPs used in this work, resulting in 15 individual uncertainty propagation curves across the temperature range. Similarly, Figure 15c presents the n = 3 scheme for all possible combinations of the six HTFPs, yielding 20 distinct configurations.

Several studies have therefore focused on identifying an optimal number of HTFPs and reference temperatures that minimize the overall uncertainty across the target temperature range. The appropriate selection of both the number and the specific combination of HTFPs in RPRT-based scale realization depends on several factors—including the temperature region of interest, the available measurement capability, and the intended application—while also enabling the minimization of uncertainty within a specific temperature interval of practical relevance.

**Figure 16:**
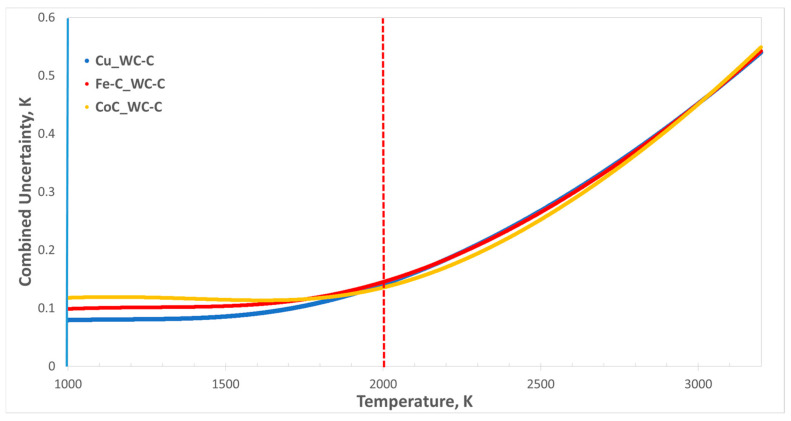
Example of uncertainty analysis for the n = 2 scheme, showing the selected optimal HTFP pairs (Cu/WC–C, Fe–C/WC–C, and Co–C/WC–C) that achieve low combined uncertainty across the 1000–2000 K range.

The appropriate selection of both the number and the specific combination of HTFPs in RPRT-based scale realization depends on several factors—including the temperature region of interest, the available measurement capability, and the intended application—while also enabling the minimization of uncertainty within a specific temperature interval of practical relevance. According to the *MeP*-K, traceability for the calibration of thermocouples in contact thermometry above the copper fixed point must be established using radiation thermometry. Most industrial and metrological applications of thermocouples operate within the temperature range from the copper point up to approximately 2000 K. As demonstrated in [29], scale realizations based on the *n* = 2 scheme provide a favorable compromise between interpolation and extrapolation, offering a balanced uncertainty distribution over a wide temperature range, including acceptable uncertainty under moderate extrapolation. For example, uncertainty analysis for the *n* = 2 scheme shows that the selected optimal HTFP pairs (Cu/WC–C, Fe–C/WC–C, and Co–C/WC–C) achieve low combined uncertainty across the 1000–2000 K range. By selecting an appropriate combination of HTFPs, the combined standard uncertainty can be maintained at a low level and exhibits a smooth, monotonic increase across the realization range, as illustrated in Figure 16. Compared with n = 1 realizations, this approach significantly reduces the uncertainty growth away from the reference temperature, while avoiding the pronounced extrapolation effects that can arise outside the interpolation range when a larger number of fixed points is employed. The resulting uncertainty behavior is particularly well suited to calibration tasks in the copper-point to 2000 K range, where the uncertainty contribution from interpolation remains dominant and well controlled. This temperature interval is also of primary importance for high-temperature industrial applications, especially in the glass and metal processing sectors, where stable traceability and predictable uncertainty behavior are critical for process control and quality assurance.

**Figure 17:**
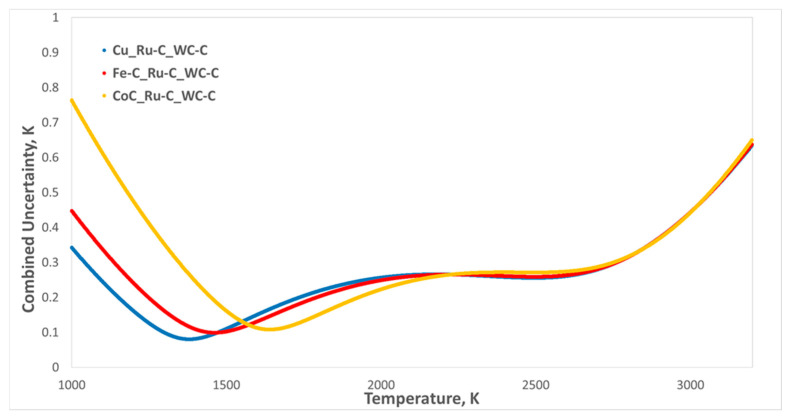
Example of uncertainty analysis for the n = 3 scheme, showing the selected optimal HTFP pairs (Cu/Ru–C/WC–C, Fe–C/Ru–C/WC–C, and Co–C/Ru–C/WC–C) that achieve low combined uncertainty across the 2500–3000 K range.

For temperature ranges around 2500–3000 K—which are of primary interest for blackbody-based photometry and spectrophotometry employing large-area, high-temperature, high-emissivity blackbodies, as well as for metal–carbide–carbon peritectic fixed points such as MoC–C and TiC–C—an alternative optimization strategy is required [60,61]. In this regime, applying the n = 3 scheme with the selected HTFPs provides an optimal uncertainty distribution over the temperature range of interest. As shown in Figure 17, selecting HTFPs from the used combinations—Cu/Ru–C/WC–C, Fe–C/Ru–C/WC–C, or Co–C/Ru–C/WC–C—allows a low and smooth combined uncertainty across 2500–3000 K to be achieved. This approach enables improved control of extrapolation uncertainties at the upper end of the scale while remaining fully consistent with the principles of RPRT-based scale realization.

Overall, these considerations highlight that the optimal strategy for RPRT-based scale realization is inherently application-dependent and requires a careful balance between interpolation range, extrapolation behavior, and practical constraints. The choice of the number and type of HTFPs should therefore be guided not only by achievable uncertainty levels, but also by the intended temperature range, measurement purpose, and available experimental resources. In this context, having a flexible and transparent analysis framework is essential for evaluating and comparing different realization schemes on a consistent metrological basis.

## 8. Conclusions

An open-source software, MultiFixRadSoft, providing a comprehensive and reliable framework for the realization of thermodynamic temperature scales based on RPRT and ITS-90 routines is presented. By integrating data preparation, phase change curve analysis, correction procedures, scale realization, and uncertainty evaluation within a unified platform, the software enables consistent and traceable evaluation of thermodynamic temperature based on HTFP measurements in accordance with metrological best practice.

The performance of MultiFixRadSoft was evaluated using experimental data obtained during the practical implementation of RPRT, employing a high-temperature furnace and a linear pyrometer. The dataset comprised measurements from several HTFP cells, including Cu, Fe–C, Co–C, Pd–C, Ru–C, and WC–C. Using commonly accepted algorithms, the software reliably identified the characteristic melting parameters—namely, the POI, LP, and melting range—as well as the freezing point for Cu.

During the subsequent correction stages, the software applied SSE, linearity, emissivity, and temperature drop corrections. The resulting corrected signals were identical to those obtained by manual analysis performed within the Real-K project. Refitted Sakuma–Hattori interpolations yielded thermodynamic temperature scales with associated uncertainties in agreement with previously published results [17,18], thereby validating the correctness of the implemented algorithms and uncertainty framework.

The built-in visualization and reporting tools enabled real-time assessment of fit quality and uncertainty propagation, ensuring a transparent and traceable workflow from raw phase change curve data to the final thermodynamic temperature realization. The automated uncertainty analysis, fully compliant with the *MeP*-K, significantly reduces analysis time while minimizing the risk of manual calculation errors.

One of the primary objectives of the MultiFixRad project is to support the development of low-cost interpolation radiation thermometers and the dissemination of radiation temperature scales based on these devices and RPRT routines, particularly for emerging NMIs and DIs. As shown by Lowe [31], such instruments can achieve uncertainties comparable to primary standards, with traceability to the kelvin above 1300 K, consistent with proposed revisions to the *MeP*-K.

The development of MultiFixRadSoft represents a collaborative outcome of the MultiFixRad project aimed at supporting emerging NMIs and promoting wider adoption of *MeP*-K-based thermometry. To ensure long-term accessibility and continued development, the source code will be made publicly available under an open-source license, together with documentation and example datasets. It is worth noting that the software has already been successfully employed for the approximation of the radiation temperature scale in the middle temperature range (approximately 400 K to 1200 K), using a series of ITS-90-defined fixed points, including indium (In), tin (Sn), zinc (Zn), aluminum (Al), silver (Ag), and copper (Cu), together with a homemade InGaAs-based radiation thermometer [62,63]. Future (ongoing) developments will include extended support for LabVIEW and MATLAB implementations, the integration of additional correction modules, and further enhancements to the uncertainty analysis framework. Additionally, a dedicated module is currently under development to ensure that the software output format supports seamless integration into Digital Calibration Certificates (DCC) workflows, enabling traceability, interoperability, and compliance with modern digital metrology practices. The development teams at TÜBİTAK UME, RISE, and JV will continue to maintain and support the platform, encouraging collaboration and contributions from the international thermometry community.

## Figures and Tables

**Table 1:** Current status of high-temperature thermodynamic fixed points, including assigned reference values and associated uncertainties.

Reference Point	Melting Temperature, K			
	POI	U_POI_	LP	U_LP_
Fe-C (T)	1426.92	0.14	1427.02	0.16
Co-C (T)	1597.39	0.13	1597.48	0.14
Pd-C (T)	1765.05	0.16	1765.18	0.18
Pt-C (T)	2011.43	0.18	2011.50	0.22
Ru-C (T)	2226.99	0.24	2227.08	0.24
Re-C (T)	2747.84	0.35	2747.91	0.44
WC-C (T)	3020.85	0.40	3020.92	0.40
	**Freezing point, K**			
Cu (T)	1357.802		U = 0.08	

**Table 2:** The POI values of the used HTFPs.

HTFPs	POI, K			
	CCT WG	Cubic Interpolation	Average	(Max − Min)/2
Fe-C (T)	1426.741	1426.728	1426.735	0.007
Co-C (T)	1597.374	1597.390	1597.382	0.008
Pd-C (T)	1764.970	1764.963	1764.967	0.004
Ru-C (T)	2226.724	2226.699	2226.712	0.013
WC-C (T)	3020.360	3020.365	3020.363	0.003

**Table 3:** The LP values of the used HTFPs.

HTFPs	Liquidus, K			
	Fraction	Intersection	Average	(Max − Min)/2
Fe-C (T)	1426.848	1426.838	1426.843	0.005
Co-C (T)	1597.545	1597.537	1597.441	0.004
Pd-C (T)	1765.076	1765.053	1765.065	0.007
Ru-C (T)	2226.783	2226.773	2226.778	0.005
WC-C (T)	3020.404	3020.394	3020.399	0.005

**Table 4:** The corrections applied to all HTFP signals used in this work.

HTFPs	Corrections, mK			
	SSE Correction	Linearity Correction	Emissivity Correction	Temperature Drop Correction
Cu	9	0.1	25	1.3
Fe-C	10	4	28	1.5
Co-C	12	5	35	2.4
Pd-C	14	10	42	3.6
Ru-C	22	16	67	9
WC-C	39	29	124	31

**Table 5:** Uncertainty budget for each HTPF used in this study for the practical implementation of the RPRT.

Uncertainty Component	Fe–C, K	Co–C, K	Pd–C, K	Ru–C, K	WC–C, K
Gain Ratio	0.009	0.012	0.014	0.022	0.041
Calibration of the radiometer	0.066	0.110	0.167	0.233	0.562
Linearity of radiometer	0.014	0.017	0.021	0.034	0.062
Ambient Temperature	0.001	0.001	0.001	0.002	0.004
The drift of the radiometer since calibration	0.006	0.007	0.009	0.014	0.025
SSE	0.018	0.022	0.028	0.045	0.083
Alignment of radiometer	0.018	0.022	0.028	0.045	0.083
Determination of POI	0.035	0.010	0.006	0.003	0.009
Stability of source	0.031	0.008	0.006	0.005	0.004
Combined Uncertainty, (*k* = 1)	0.09	0.12	0.18	0.25	0.58
**Expanded Uncertainty, (*k* = 2)**	**0.17**	**0.23**	**0.35**	**0.49**	**1.16**

**Table 6:** Uncertainty table for ITS-90 scale realization on Cu-point.

Source of Uncertainty	Uncertainty Contribution, K
Impurity	0.005
Emissivity	0.009
Temperature drop	0.003
Cu–Plateau Determination	0.011
Repeatability	0.025
Wavelength	0.000
Repeatability	0.000
Short time Drift	0.006
Out-of-band transmission	0.000
Size-of-source	0.005
Non-linearity	0.015
Drift	0.026
Combined Uncertainty (*k* = 1), U	0.05
**Expanded Uncertainty (*k* = 2), U**	**0.09**

## Data Availability

The MultiFixRadSoft open-source code (v2.0.0) will be released under a Clear BSD license via GitHub and the Python Package Index (PyPI), and the MATLAB version together with full documentation will be made publicly available on Zenodo. The software and documentation will be published as a citable reference linked to the article DOI.
